# Real-World Outcomes of Adolescents and Young Adults with Diffuse Large B-Cell Lymphoma: A Multicenter Retrospective Cohort Study

**DOI:** 10.1089/jayao.2023.0095

**Published:** 2024-04-02

**Authors:** Denisse Castro-Uriol, Ligia Rios, Daniel Enriquez-Vera, Jacqueline Montoya, Thanya Runciman, Sandra Alarcón, Arturo Zapata, Eddy Hernández, Esmeralda León, Luis Malpica, Bryan Valcarcel

**Affiliations:** ^1^ Departamento de Oncología y Radioterapia, Hospital Nacional Edgardo Rebagliati Martins, Lima, Peru.; ^2^ Centro de Medicina de Precisión, Instituto de Investigación, Universidad de San Martín de Porres, Lima, Perú.; ^3^ Unidad de Oncología Pediátrica y del Adolescente, Departamento de Oncología y Radioterapia, Hospital Nacional Edgardo Rebagliati Martins, Lima, Perú.; ^4^ Division of HTLV-1/ATL Carcinogenesis and Therapeutics, Joint Research Center for Human Retrovirus Infection, Kagoshima University, Kagoshima, Japan.; ^5^ Departamento de Oncología Pediátrica, Instituto Nacional de Enfermedades Neoplásicas, Lima, Peru.; ^6^ Departamento de Oncología y Radioterapia, Hospital Nacional Guillermo Almenara Irigoyen, Lima, Peru.; ^7^ Unidad de Oncología Pediátrica y del Adolescente, Departamento de Oncología y Radioterapia, Hospital Nacional Guillermo Almenara Irigoyen, Lima, Perú.; ^8^ Division of Cancer Medicine, Department of Lymphoma and Myeloma, The University of Texas MD Anderson Cancer Center, Houston, Texas, USA.; ^9^ Department of Epidemiology, Milken Institute School of Public Health, The George Washington University, Washington, District of Columbia, USA.

**Keywords:** young adults, adolescents, diffuse large B cell lymphoma, outcomes, cohort studies

## Abstract

**Purpose:**

Patients with diffuse large B-cell lymphoma (DLBCL) are typically treated with rituximab, cyclophosphamide, doxorubicin, vincristine, and prednisone (R-CHOP). However, a standard of care for managing adolescents and young adults (AYAs) with DLBCL is lacking. We examine treatment approaches and outcomes of this population.

**Methods:**

We included 90 AYAs (15–39 years) diagnosed with DLBCL between 2008 and 2018 in three tertiary centers in Peru. Overall response rates (ORR) were available for all patients. Overall survival (OS) and progression-free survival (PFS) rates were estimated using the Kaplan–Meier method.

**Results:**

The median age at diagnosis was 33 years, 57% were males, 57% had good performance status (Lansky/Karnofsky ≥90), and 61% were diagnosed with early-stage disease (Ann Arbor stages I–II). R-CHOP (*n* = 69, 77%) was the most frequently used first-line regimen, with an ORR of 91%. With a median follow-up of 83 months, the 5-year OS and PFS among all patients were 79% and 67%, respectively. Among the patients who received R-CHOP, the 5-year OS and PFS were 77% and 66%, respectively. Of the 29 (32%) patients with relapsed/refractory (R/R) disease, 83% received second-line treatment and only 14% underwent consolidation therapy with autologous transplantation. The 3-year OS for R/R DLBCL was 36%.

**Conclusion:**

Our data show that AYAs with DLBCL who received conventional therapy had comparable outcomes to those observed in studies conducted among the adult population. However, the prognosis for AYAs with R/R disease was dismal, indicating the unmet need for developing and increasing access to novel treatment modalities in AYAs.

## Introduction

Lymphoma is one of the most common malignancies among adolescents and young adults (AYAs) and contributes substantially to the worldwide cancer burden in this population.^1^ Among non-Hodgkin lymphoma (NHL) subtypes, diffuse large B-cell lymphoma (DLBCL) and Burkitt lymphoma are the two most prevalent after Hodgkin lymphoma in AYAs.^2^

Compared to the pediatric and adult populations, the management of aggressive lymphomas in AYAs lacks standardization. Various treatment regimens have been proposed by pediatric and adult working groups for AYAs with DLBCL.^3^ Pediatric centers typically use the Berlin-Frankfurt-Mü (BFM) and the French-American-British/Lymphome Malins de Burkitt (FAB/LMB) protocols, while adult centers administer shorter anthracycline-based regimens such as rituximab, cyclophosphamide, vincristine, doxorubicin, and prednisone (R-CHOP).^4^ BFM and FAB/LMB can achieve event-free survival (EFS) of 82% and 87%, respectively.^5,6^ In contrast, the R-CHOP for adult population, 6-year EFS of 60%–80%.^7^

Population-based studies in DLBCL have shown differences in treatment approaches, disease biology, and comorbidities between age groups. There is a higher 5-year overall survival in children (90.7%) compared to AYAs (81.7%).^8^ However, survival outcomes between AYAs and adults have reported disparate results in the literature.^6,7,9–12^ One study did not find differences between age groups (2-year OS rate of 68.5% in AYAs and 78.7% in adults, *p* = 0.193).^9^ On the contrary, a different study reported that the 5-year relative survival rates (RSR) were 79% for AYAs and 59% for adults with DLBCL, respectively.^12^

The limited representation of the AYAs population in clinical trials has hindered the establishment of a standard of care.^13–17^ Thus, real-world data may enhance our understanding of existing treatment practices to inform the design of further studies aimed at developing a standardized protocol for this age group. This study reports our experience regarding treatment approaches and survival outcomes among Latin American AYAs diagnosed with DLBCL in three academic cancer centers.

## Materials and Methods

### Study design and population

We designed a multicenter retrospective cohort study of patients diagnosed with DLBCL between January 2008 and December 2018, with follow-up (FU) until December 2021. Three tertiary cancer centers located in Lima, the capital city of Peru, participated in this study: Hospital Nacional Edgardo Rebagliati Martins; Instituto Nacional de Enfermedades Neoplasicas; and Hospital Nacional Guillermo Almenara Irigoyen. These centers concentrate over 73% of the cancer care in the country,^18^ providing a fair representation of cancer care.

The patients were identified through the electronic medical record system at each participating center. Medical records were manually reviewed, and clinical data were abstracted in a standardized form. We included patients aged 15–39 years according to the definition of AYAs working groups,^1,13^ those with an anatomopathological diagnosis of DLBCL according to the World Health Organization Classification,^14^ and at least received three cycles of chemotherapy. We excluded individuals with lost medical records or with insufficient data for pathological characterization. The patients were linked to The National Registry of Identification and Civil Status (RENIEC, in Spanish) database to confirm their vital status.

### Study variables

We collected data for the following demographic and clinical variables: sex, age, place of residence, performance status, presence of B symptoms, histological subtype, clinical stage, presence of extranodal disease, and treatment response. “Lima province,” refers to patients who come from the capital city of Lima, and “Other provinces” refers to patients who come from outside the capital city of Lima or countryside.

We stratified patients into early (15–24 years) and late AYAs (25–39 years). Performance status was evaluated using the Lansky and Karnosky scales; the former was performed in patients aged <16 years, and the latter in those aged ≥16 years. A good performance status was defined as a Lansky/Karnofsky ≥90%. We used the Murphy staging system for lymphoma patients aged <16 years and the Ann Arbor staging system for those aged ≥16 years.^19,20^ We defined treatment abandonment as patients who were lost to FU after completing three cycles of chemotherapy.

### Treatment regimens

The treatment regimen used for the management of DLBCL among AYAs in Peru is not standardized and follows local institutional practices. At Hospital Nacional Edgardo Rebagliati Martins, patients aged 15–17 years received the pediatric FAB/LMB regimen, which categorizes patients based on a risk stratification approach.^3^ The low-risk group underwent two cycles of cyclophosphamide, vincristine, prednisone, and doxorubicin. In the intermediate-risk group, a low-dose cyclophosphamide, vincristine, and prednisone (COP), followed by an induction therapy of cyclophosphamide, vincristine, prednisone, and doxorubicin, along with high-dose methotrexate (HD-MTX 3 g/m^2^, COPADM) was administered. Then received two consolidation cycles with cytarabine and methotrexate.

For the high-risk group, a low-dose of COP, followed by an induction therapy of two cycles of COPADM (HD-MTX 8 g/m^2^), and a consolidation high-dose continuous cytarabine along with etoposide was administered. Rituximab was incorporated into all cases with clinical stage III–IV.^6^ On the contrary, older patients received either the CHOP regimen (cyclophosphamide, vincristine, doxorubicin, and prednisone) or etoposide-based regimens (dose-adjusted etoposide, prednisone, vincristine, cyclophosphamide, and doxorubicin [EPOCH] or etoposide, prednisone, vincristine, cyclophosphamide, and doxorubicin [CHOEP]) with or without rituximab (R) every 3 weeks.^21^ At the Instituto Nacional de Enfermedades Neoplásicas and the Hospital Nacional Guillermo Almenara all patients received CHOP or etoposide-based regimens with or without R.

Consolidation with radiotherapy was indicated after first-line treatment when patients had early-stage disease (stages I–II) or advanced-stage disease (stages III–IV) with a bulky mass. In addition, radiotherapy could also be utilized in the salvage or palliative care setting. For consolidation, a three-dimensional conformal radiation therapy (3D CRT) approach was adopted at a dose of 30–36 Gy, while a 3D CRT technique at dose of 40–55 Gy was given for salvage or palliative treatment.

The best treatment response was identified using the Cheson criteria and was classified as complete response (CR), partial response (PR), stable disease (SD), or progressive disease (PD) as previously described.^22^ Overall response rate (ORR) was defined as the proportion of patients with CR or PR.^23^

### Study endpoints

The study endpoints were overall survival (OS), progression-free survival (PFS) rates, and ORR. OS was defined as date of first-line treatment to death from any cause. PFS was defined as time from first-line treatment to relapse, disease progression, or death from any cause, whichever occurred first.

### Statistical analysis

We summarized demographic and clinical features with descriptive statistics. Median FU was estimated with the reverse Kaplan–Meier method. Survival probabilities were estimated using the Kaplan–Meier methods, and the log-rank test was used to identify differences between patient subgroups. Cox regression analyses were performed to evaluate the association of clinical and demographic factors with OS and PFS. Our models were adjusted to typical characteristics associated with mortality or recurrence (age, sex, performance status, extranodal involvement, clinical stage, and treatment regimen), and to demographic variables deemed of relevance (place of residence), regardless of their statistical significance in the univariate analysis.^9,10^ The proportional hazard assumption was checked with the goodness-of-fit test and with log(-log [survival]) plots. There was no violation of the hazard assumption. We present our results with Hazard ratios (HRs) and 95% confidence intervals (CIs). *p*-Values <0.05 were considered statistically significant. Analyses were conducted in R version 4.1.1.

### Ethical statement

The Institutional Review Board at each participating center approved this study. Patient-identifiable data were censored, and each patient was assigned a numeric code (i.e., 1, 2, 3, and so on).

## Results

**FIG. 1.**
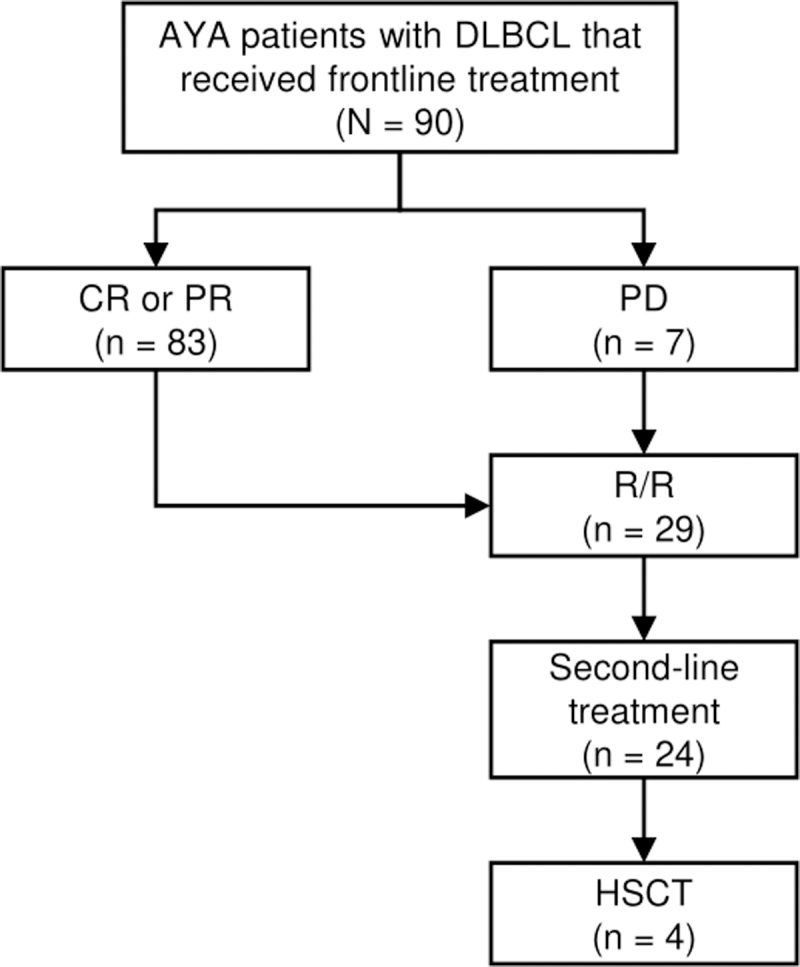
Flowchart of AYAs with DLBCL who received a first-line regimen. AYAs, adolescents and young adults; DLBCL, diffuse large B-cell lymphoma.

We identified 90 patients diagnosed with DLBCL who received a first-line regimen during 2008–2018 (Fig. 1). Table 1 shows the clinical features of the overall cohort. The median age at diagnosis was 33 years. Most of them were late AYAs (25–39 years, 91%), had a slight male predominance (57%), and were from “Other provinces” (54%). Most patients had good performance status (Lansky or Karnofsky ≥90%, 57%), presented with B symptoms (58%), had extranodal involvement (49%), and early-stage disease (61%) at diagnosis. Treatment abandonment was reported for 16% (*n* = 14/90) of the overall population.

**Table 1.** Demographic and Clinical Features of Adolescents and Young Adults with Diffuse Large B-Cell Lymphoma

Characteristics	All patients, No. (%)
No. of patients	90
Median age at diagnosis, years	33
Age group	
15–24	8 (9)
25–39	82 (91)
Males	51 (57)
Place of residence	
Lima province	41 (46)
Other provinces	49 (54)
Performance status^a^	
<90	39 (43)
≥90	51 (57)
Nodal involvement	70 (78)
Extranodal involvement	44 (49)
B symptoms	52 (58)
Clinical stage	
I–II	55 (61)
III–IV	35 (39)
Frontline regimen	
R-CHOP	69 (78)
R-EPOCH	7 (8)
CHOP	5 (6)
R-CHOEP	3 (3)
CHOEP	3 (3)
FAB/LMB	2 (2)
Unknown	1
Radiotherapy^b^	37 (41)
Relapse/refractory	29 (32)
Second-line regimen	24 (27)
HSCT after second line	4 (4)
Treatment abandonment	14 (16)

^a^ Performance status was evaluated by Lansky or Karnofsky scales.

^b^ Radiotherapy treatment as a consolidation or palliative treatment.

CHOP, cyclophosphamide, vincristine, doxorubicin and prednisone; CHOEP, cyclophosphamide, vincristine, doxorubicin, etoposide, and prednisone; FAB/LMB, French-American-British/Lymphome Malins B regimen; HSCT, hematopoietic stem cell transplantation; R-CHOP, rituximab, cyclophosphamide, vincristine, doxorubicin, and prednisone; R-CHOEP, rituximab, cyclophosphamide, vincristine, doxorubicin, etoposide and prednisone; R-EPOCH, rituximab, etoposide, prednisone, vincristine, cyclophosphamide, and doxorubicin.

### First-line treatment

The most common first-line approach was R-CHOP (78%). The R-EPOCH regimen was the second most common in 8% of the patients, while only 2% of AYAs receiving the FAB/LMB protocol (Table 1). Table 2 describes the first-line chemotherapy regimen and treatment responses. Of all AYAs, the ORR was 92% (*n* = 83/90), and 8% (*n* = 7/90) had PD. Patients who received R-CHOP achieved an ORR of 91% (CR = 68%). Although limited in sample size, patients receiving R-EPOCH, R-CHOEP, CHOEP, or FAB/LMB regimens had an ORR of 100%.

**Table 2.** First-Line Treatment Responses of Adolescents and Young Adults with DIFFUSE Large B Cell Lymphoma

	Treatment responses, No. (%)				
	ORR (CR+PR)	CR	PR	SD	PD
Overall, row %	83 (92)	61 (68)	22 (24)	—	7 (8)
Frontline regimen, row %					
R-CHOP	63 (91)	47 (68)	16 (23)	—	6 (9)
R-EPOCH	7 (100)	2 (29)	5 (71)	—	—
R-CHOEP	3 (100)	3 (100)	—	—	—
CHOP	4 (80)	4 (80)	—	—	1 (20)
CHOEP	3 (100)	2 (67)	1 (33)	—	—
FAB/LMB	2 (100)	2 (100)	—	—	—
Unknown		1	—	—	—

CR, complete response; ORR, overall response rate; PD, progressive disease; PR, partial response; SD, stable disease.

A total of 31 patients received consolidation radiotherapy, 23 (74%) with early-stage disease, and 8 (26%) with advanced-stage disease.

### Survival outcomes

**FIG. 2.**
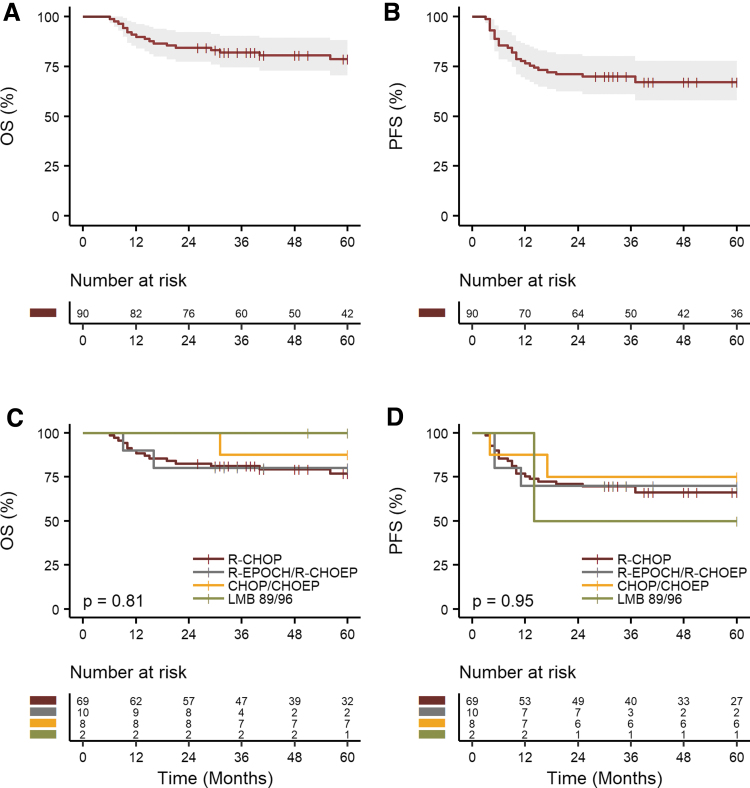
Kaplan–Meier curves for OS **(A)**, PFS **(B)**, for the overall AYAs population with DLBCL. OS **(C)** and PFS **(D)** according to chemotherapy regimens of AYAs. OS, overall survival; PFS, progression-free survival.

After a median FU of 83 months (95% CI 76–104), 18 patients were reported as deceased after first-line treatment. The 5-year OS and PFS were 79% (95% CI 71%–88%) and 67% (95% CI 58–78) for all patients, respectively (Fig. 2A, B). The median OS and PFS were not reached. The 5-year OS and PFS for patients that received R-CHOP (*n* = 69) were 77% (95% CI 67–88) and 66% (95% CI 56–78), respectively.

For the subgroup of patients (*n* = 10) who received either R-EPOCH or R-CHOEP, the 5-year OS and PFS rates were 80% (95% CI 59–100) and 70% (95% CI 47–100), respectively. Patients treated with CHOP or CHOEP (*n* = 8) had 5-year OS and PFS rates of 88% (95% CI 67–100) and 75% (95% CI 50–100), respectively (Fig. 2C, D). The multivariable Cox analysis did not reveal any statistically significant OS or PFS advantage for any regimen when compared to R-CHOP (Supplementary Table S1).

Supplementary Figure S1 illustrates the 5-year OS and PFS rates by demographic factors such as age group (15–24 years vs. 25–39 years), biological sex (male vs. female), place of residence (Lima province vs. Other provinces), performance status (Lansky/Karnofsky scale <90 vs. ≥90), and clinical stage (stages I–II vs. III–IV). Overall, there was no statistically significant difference in survival outcomes by demographic and clinical variables. Supplementary Table S1 shows that no variable was a significant predictor for OS or PFS in the univariable or multivariable Cox regression analyses.

### Treatment approaches and outcomes of R/R disease

Thirty-two percent (*n* = 29) of the population had R/R disease, 22 after achieving a CR or PR, and 7 with PD (Fig. 1 and Supplementary Table S2). Of them, 83% (*n* = 24/29) received second-line treatment (Supplementary Table S2). Five patients were not eligible for second-line treatment because of poor performance status and only received palliative care. In the salvage setting, the preferred regimen was rituximab, ifosfamide, carboplatin, and etoposide (R-ICE) in 46% of the patients, followed by rituximab, etoposide, methylprednisone, cytarabine, and cisplatin (R-ESHAP) in 34% of the patients. Consolidation with hematopoietic stem cell transplantation (HSCT) after salvage second-line treatment was performed in 17% (*n* = 4/24) of R/R patients (Supplementary Table S2). Radiotherapy was used as palliative care in six patients.

**FIG. 3.**
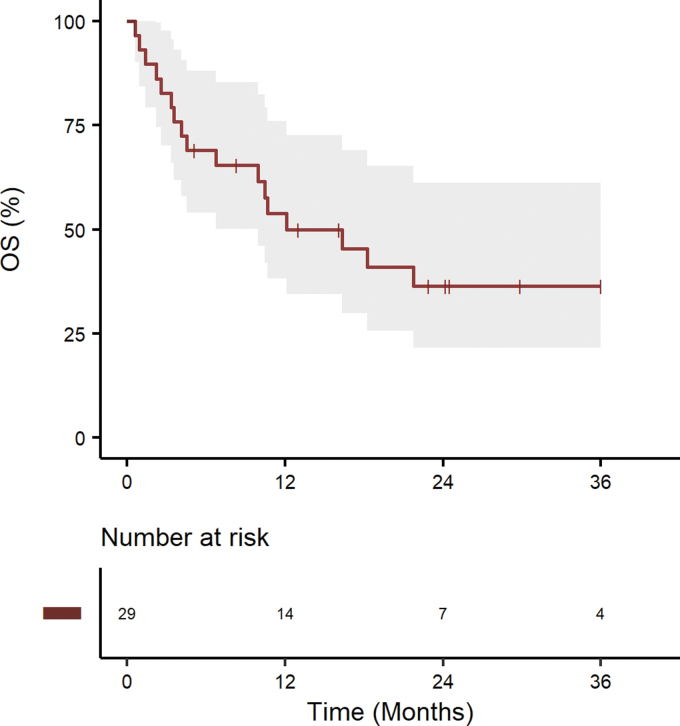
Kaplan–Meier curves in R/R for OS in AYAs with DLBCL. R/R, relapsed/refractory.

Figure 3 illustrates the Kaplan–Meier curves for R/R disease. With a median FU of 30 months (95% CI 22.8–61.2), the 3-year OS rate was 36% (95% CI, 22%–59%) and the median OS was 12 months (95% CI, 7 months–not reached).

## Discussion

Few studies have described real-world treatment patterns and outcomes of AYAs with DLBCL in Latin America. We conducted a multicenter cohort study in three academic cancer centers in Peru. Our study identified that AYAs with DLBCL were mostly treated with CHOP-like regimen (with or without rituximab), and their survival outcomes were comparable to those observed in previous trials among the adult population. However, we found a dismal prognosis for those with R/R disease, highlighting an unmet need to develop approaches and increased access to novel treatments to improve outcomes in this setting.

Although our study does not aim to compare the survival between AYAs and the adult population, it seems to be similar between those groups. Studies have reported conflicting results regarding survival outcomes between AYAs and the adult populations. Similar to our study, other studies have not found differences between both groups. Suzuki et al. studied 798 patients with DLBCL where 95% were adults (*n* = 756) and 5% were AYAs (*n* = 42 patients). The median FU was 23.6 months and found that survival outcomes were similar to adults aged 40–60 years. The 2-year PFS rates in AYAs compared to adults were 61.9% and 65.3% (*p* = 0.9191), and the 2-year OS rates in AYAs were 68.5% and 78.7% (*p* = 0.193), respectively.^9^

Similarly, Coso et al. matched 54 AYAs with 162 adults, and there were no differences between groups regarding EFS (68% vs. 69%, *p* = 0.900) and OS (75% vs. 73%, *p* = 0.800).^10^ A more recent study by Başcı et al. found that the median PFS and OS rates were not reached without any difference between age groups (18–39 years, *n* = 40 vs. ≥40 years, *n* = 80).^11^ The 5-year OS rate for AYAs and non-AYAs were 75% and 65% (*p* = 0.700), respectively.

Other studies have demonstrated improved outcomes in AYAs compared to adults.^7,12^ In the MabThera International Trial found 6-year OS rates of 80% versus 90% in AYAs with DLBCL treated with chemotherapy vs chemotherapy plus rituximab, respectively.^7^ In the United States, Blum et al. reported 2- and 5-year RSR of 82.7% and 79% in AYAs with DLBCL, and 65% and 59% in adults with DLBCL, respectively.^12^ It is likely that comorbidities, age-related factors, and tumor biology may explain these disparities between age groups.^24^ Other studies reported that AYAs hold poor outcomes probably related to the inclusion of primary mediastinal B cell lymphoma patients.^2,6^ Likewise, others have reported worse outcomes in AYAs as result of increased susceptibility to therapy-related toxicity, suboptimal compliance to prescribed regimens, and lower rates of clinical trial enrollment.^13,16,25^

It is a common clinical practice to allot early AYAs (15–24 years) to pediatric approaches while late AYAs (25–39 years) to adult regimens.^26–28^ Pediatric regimens such as the BFM and FAB/LMB are the most used,^3^ with favorable outcomes in AYAs with DLBCL.^10^ The EFS for these regimens are 82% and 87%, respectively.^5,6^ In contrast, in adult patients, the standard regimen for DLBCL is R-CHOP, achieving a 6-year EFS of 60%–80%.^7^ In a recent publication, Gupta et at. analyzed 176 AYAs with B cell lymphomas, where 73.3% had DLBCL. Patients who received pediatric protocols had better EFS (82.3 vs. 66.7%, *p* = 0.02) and OS (85.5% vs.71.1%, *p* = 0.03) than those receiving adult protocols.^29^

Interestingly, a higher number of patients receiving adult protocols also received rituximab compared to patients on pediatric protocols.^29^ In our study, we found that most patients were managed with adult protocols (98%). We also found that those who received intensive regimens achieved higher ORR. However, direct comparisons are limited to the low sample size in each treatment modality. Therefore, these findings need to be confirmed prospectively and with larger sample sizes.

In this study, we did not find differences in survival between early and late AYAs, probably related to the low number of patients in the early AYAs group. Hohloch et al. allocated patients in three age groups (18–25, 26–30, and 31–35 years).^30^ The majority of patients had DLBCL (71%). There were no differences in EFS and OS rates between age groups (EFS: 69%, 71%, and 72%, *p* = 0.955; OS: 89%, 85%, and 88%, *p* = 0.527; respectively).^30^ Also, there was no difference in the three age groups regarding the rituximab use.

We found that patients living in “Other province” had comparable survival outcomes to those living in Lima. Several reports have suggested that patients who reside in places far away from health care centers have worse outcomes.^9,31,32^ Our findings might be explained by how the Peruvian public health care system works which provides free health care access with social support (e.g., coverage for travel expenses and lodging during their cancer care); and rapid access to medical care with appointments scheduled in a timely manner, especially for those deemed as high-risk.

Approximately, one-third of our population (34%) experienced R/R disease which is higher compared to previous studies.^11,33^ Cairo et al. reported that 9.4% (*n* = 104) of individuals had R/R disease,^33^ while Başcı et al. found that 22.4% (*n* = 9) had R/R after first-line treatment.^11^ The different study design and patient populations between our cohort and these two studies may explain the higher rate of R/R disease. Cairo et al. conducted a multicentric trial, whereas Başcı et al. performed a single-center cohort study. Furthermore, the typical more stringent eligibility criteria in the multicentric trial and the potential for loss to FU in the single-center study may suggest an underestimation of R/R disease in these experiences.^11,33^

The 3-year OS rate of 36% of R/R patients in our cohort is similar to a previous study.^33^ Cairo et al. reported 1- and 2-year OS rates of 31.5% and 22.3%, respectively, for AYAs with B cell NHL who relapsed or progressed to first-line chemotherapy regimens.^33^ These outcomes underscore the importance of identifying potential biomarkers to identify patients at higher risk for R/R disease and develop novel treatment strategies to improve the outcomes of these patients. Notably, only a small proportion (4%) of our patients underwent HSCT after second-line treatment. In Peru, access to HSCT is limited and few centers have the infrastructure to perform this treatment modality. The low percentage of HSCT use in our cohort represent an unmet need to increase access to transplantation in Peru. Similarly, novel therapies such as chimeric antigen receptor T cell therapy, is not yet available in Peru.

This study has limitations. We could not compare pediatric to adult regimens because only a small proportion of patients received pediatric protocols (2%). This is partially explained by the heterogeneity and the lack of consensus on the optimal treatment regimen for AYAs. Treatment-related toxicities, comorbidities, and second-line treatment responses were not consistently recorded in the medical records, thus, we were unable to estimate the effect of these factors on the observed outcomes. Because of the limited availability of HSCT in Peru and low sample size of patients who received this treatment in our cohort (*n* = 4), the assessment of post-transplantation survival outcomes among R/R patients was not conducted. We also lacked data on HIV status because screening is not routinely performed in some centers.

Finally, genetic profiling and tumor molecular assessment are not routinely performed in Peruvian centers, thus, we could not evaluate their impact on survival outcomes. Despite these limitations, this multicenter study includes a longer FU period and larger sample size compared to previous real-world reports on AYAs with DLBCL.^9,11^

## Conclusion

This multicentric study provides real-world data on the treatment patterns and outcomes of AYAs diagnosed with DLBCL in Peru. Our findings suggest similar outcomes to those reported internationally after first-line treatment. Although we found good outcomes with less intensive protocols, future prospective studies should determine the best treatment approach for this patient population. Patients who experienced R/R disease had a dismal prognosis, with a high mortality rate within 2 years of R/R diagnosis, underscoring a significant unmet need in this setting. Greater efforts should be made to identify high-risk patients and increase access to novel treatments to improve outcomes in R/R patients.

## Supplementary Material

Supplemental data

Supplemental data

Supplemental data

## References

[1] Alvarez EM, Force LM, Xu R, et al. The global burden of adolescent and young adult cancer in 2019: A systematic analysis for the Global Burden of Disease Study 2019. Lancet Oncol 2021;23(1):27–52; doi: 10.1016/s1470-2045(21)00581-7 34871551 PMC8716339

[2] Hochberg J, El-Mallawany NK, Abla O. Adolescent and young adult non-Hodgkin lymphoma. Br J Haematol 2016;173(4):637–650; doi: 10.1111/bjh.14074 27071675

[3] Brugières L, Brice P. Lymphoma in adolescents and young adults. Prog Tumor Res 2016;43:101–114; doi: 10.1159/000447080 27595360

[4] Bouabdallah R, Coso D, Garciaz S. Clinical and biological aspects of aggressive B-cell non-Hodgkin lymphoma in adolescents and young adults. Clin Oncol Adolesc Young Adults 2015;115; doi: 10.2147/coaya.s70365

[5] Burkhardt B, Oschlies I, Klapper W, et al. Non-Hodgkin's lymphoma in adolescents: Experiences in 378 adolescent NHL patients treated according to pediatric NHL-BFM protocols. Leukemia 2011;25(1):153–160; doi: 10.1038/leu.2010.245 21030984

[6] Cairo MS, Sposto R, Gerrard M, et al. Advanced stage, increased lactate dehydrogenase, and primary site, but not adolescent age (≥15 years), are associated with an increased risk of treatment failure in children and adolescents with mature B-cell non-Hodgkin's lymphoma: results of the FAB LM. J Clin Oncol Off J Am Soc Clin Oncol 2012;30(4):387–393; doi: 10.1200/JCO.2010.33.3369 PMC3269965 22215753

[7] Pfreundschuh M, Kuhnt E, Trümper L, et al. CHOP-like chemotherapy with or without rituximab in young patients with good-prognosis diffuse large-B-cell lymphoma: 6-year results of an open-label randomised study of the MabThera International Trial (MInT) Group. Lancet Oncol 2011;12(11):1013–1022; doi: 10.1016/S1470-2045(11)70235-2 21940214

[8] Abrahão R, Ribeiro RC, Lichtensztajn DY, et al. Survival after diffuse large B-cell lymphoma among children, adolescents, and young adults in California, 2001–2014: A population-based study. Pediatr Blood Cancer 2019;66(4):e27559; doi: 10.1002/pbc.27559 30511461 PMC9423938

[9] Suzuki Y, Yano T, Suehiro Y, et al. Clinical characteristics and outcomes of diffuse large B-cell lymphoma in adolescents and young adults. Int J Hematol 2018;108(2):161–166; doi: 10.1007/s12185-018-2449-8 29605873

[10] Coso D, Garciaz S, Esterni B, et al. Large B-cell lymphomas in adolescents and young adults in comparison to adult patients: A matched-control analysis in 55 patients. Leuk Lymphoma 2014;55(8):1849–1853; doi: 10.3109/10428194.2013.858814 24160849

[11] Başcı S, Bakırtaş M, Yiğenoğlu TN, et al. The outcome of diffuse large B cell lymphoma patients in adolescent and young adult age group. J Adolesc Young Adult Oncol 2021;10(4):483–487; doi: 10.1089/JAYAO.2020.0145 33237829

[12] Blum KA, Keller FG, Castellino S, et al. Incidence and outcomes of lymphoid malignancies in adolescent and young adult (AYA) patients in the United States. Br J Haematol 2018;183(3):385; doi: 10.1111/BJH.15532 30095154 PMC6234103

[13] Smith AW, Keegan T, Hamilton A, et al. Understanding care and outcomes in adolescents and young adult with Cancer: A review of the AYA HOPE study. Pediatr Blood Cancer 2019;66(1):e27486; doi: 10.1002/PBC.27486 30294882 PMC7239374

[14] Sankaran H, Finnigan SR, McShane LM, et al. Enrollment of adolescent and young adult patients newly diagnosed with cancer in NCI CTEP-sponsored clinical trials before and after launch of the NCI National Clinical Trials Network. Cancer 2022;128(21):3843–3849; doi: 10.1002/CNCR.34402 36089859 PMC9826149

[15] Husson O, Reeve BB, Darlington AS, et al. Next step for global adolescent and young adult oncology: A core patient-centered outcome set. JNCI J Natl Cancer Inst 2022;114(4):496; doi: 10.1093/JNCI/DJAB217 34865066 PMC9002284

[16] Ferrari A, Stark D, Peccatori FA, et al. Adolescents and young adults (AYA) with cancer: A position paper from the AYA Working Group of the European Society for Medical Oncology (ESMO) and the European Society for Paediatric Oncology (SIOPE). ESMO open 2021;6(2):100096; doi: 10.1016/J.ESMOOP.2021.100096 33926710 PMC8103533

[17] Muffly L, Yin J, Jacobson S, et al. Disparities in trial enrollment and outcomes of Hispanic adolescent and young adult acute lymphoblastic leukemia. Blood Adv 2022;6(14):4085–4092; doi: 10.1182/BLOODADVANCES.2022007197 35838753 PMC9327550

[18] Ministerio de Salud -PERU. Plan Nacional de Cuidados Integrales Del Cáncer 2020–2024. n.d. Available from: https://bvs.minsa.gob.pe/local/MINSA/5341.pdf [Last accessed: July 3, 2023].

[19] Armitage JO. Staging non-Hodgkin lymphoma. CA Cancer J Clin 2005;55(6):368–376; doi: 10.3322/canjclin.55.6.368 16282281

[20] Rosolen A, Perkins SL, Pinkerton R, et al. Revised international pediatric non-Hodgkin lymphoma staging system. J Clin Oncol 2015;33:2112–2118; doi: 10.1200/JCO.2014.59.7203 25940716 PMC4461808

[21] Kahn JM, Ozuah NW, Dunleavy K, et al. Adolescent and young adult lymphoma: Collaborative efforts toward optimizing care and improving outcomes. Blood Adv 2017;1(22):1945–1958; doi: 10.1182/bloodadvances.2017008748 29296842 PMC5728148

[22] Cheson BD, Fisher RI, Barrington SF, et al. Recommendations for initial evaluation, staging, and response assessment of Hodgkin and non-Hodgkin lymphoma: The Lugano classification. J Clin Oncol 2014;32:3059–3067; doi: 10.1200/JCO.2013.54.8800 25113753 PMC4979083

[23] Smith BD, DeZern AE, Bastian AW, et al. Meaningful endpoints for therapies approved for hematologic malignancies. Cancer 2017;123(10):1689–1694; doi: 10.1002/CNCR.30622 28222220

[24] Weber T, Schmitz R. Molecular subgroups of diffuse large b cell lymphoma: Biology and implications for clinical practice. 1912;1:3; doi: 10.1007/s11912-021-01155-2 PMC8831345 35060000

[25] Burke MJ, Devidas M, Chen Z, et al. Outcomes in adolescent and young adult patients (16 to 30 years) compared to younger patients treated for high-risk B-lymphoblastic leukemia: Report from Children's Oncology Group Study AALL0232. Leukemia 2022;36(3):648–655; doi: 10.1038/S41375-021-01460-6 34725453 PMC9014378

[26] Boissel N, Auclerc MF, Lhéritier V, et al. Should adolescents with acute lymphoblastic leukemia be treated as old children or young adults? Comparison of the French FRALLE-93 and LALA-94 trials. J Clin Oncol 2003;21(5):774–780; doi: 10.1200/JCO.2003.02.053 12610173

[27] Stock W, La M, Sanford B, et al. What determines the outcomes for adolescents and young adults with acute lymphoblastic leukemia treated on cooperative group protocols? A comparison of Children's Cancer Group and Cancer and Leukemia Group B studies. Blood 2008;112(5):1646; doi: 10.1182/BLOOD-2008-01-130237 18502832 PMC2518876

[28] Rios L, Castro D, Vásquez L, et al. Survival in adolescents and young adults with B-cell non-Hodgkin's lymphoma in a referral hospital in Peru. Onkoresearch J 2022;1(1):21–26.

[29] Gupta S, Alexander S, Pole JD, et al. Superior outcomes with paediatric protocols in adolescents and young adults with aggressive B-cell non-Hodgkin lymphoma. Br J Haematol 2022;196(3):743–752; doi: 10.1111/BJH.17862 34599525

[30] Hohloch K, Zeynalova S, Held G, et al. Excellent outcome of young adults with aggressive non-Hodgkin lymphomas treated with CHOP-like regimens. Leukemia 2014;28(11):2260–2263; doi: 10.1038/LEU.2014.213 25005242

[31] Petridou ET, Dimitrova N, Eser S, et al. Childhood leukemia and lymphoma: Time trends and factors affecting survival in five Southern and Eastern European Cancer Registries. Cancer Causes Control 2013;24(6):1111–1118; doi: 10.1007/S10552-013-0188-Y 23529470

[32] Ambroggi M, Biasini C, Del Giovane C, et al. Distance as a barrier to cancer diagnosis and treatment: Review of the literature. Oncologist 2015;20(12):1378–1385; doi: 10.1634/THEONCOLOGIST.2015-0110 26512045 PMC4679078

[33] Cairo M, Auperin A, Perkins SL, et al. Overall survival of children and adolescents with mature B cell non-Hodgkin lymphoma who had refractory or relapsed disease during or after treatment with FAB/LMB 96: A report from the FAB/LMB 96 study group. Br J Haematol 2018;182(6):859–869; doi: 10.1111/BJH.15491 29984828 PMC6128751

