# Supplementary material for: Real-World Outcomes of Adolescents and Young Adults with Diffuse Large B-Cell Lymphoma: A Multicenter Retrospective Cohort Study
Source: J Adolesc Young Adult Oncol. 2024 Apr 2;13(2):323–30. doi: 10.1089/jayao.2023.0095 (PMC10998009; doi:10.1089/jayao.2023.0095)
Supplement: Supplemental data [file Suppl_TableS1.docx]

**Supplementary Table 1**. Univariate and multivariate Cox analysis for OS and PFS among all AYAs with DLBCL.

|  | **N (%)** | **OS** | | **PFS** | |
| --- | --- | --- | --- | --- | --- |
|  |  | **HR (95% CI)** | **aHR (95% CI)** | **HR (95% CI)** | **aHR (95% CI)** |
|  |  |  |  |  |  |
| Median age, years | 33 | 0.98 (0.91-1.06) | 0.94 (0.85-1.04) | 0.99 (0.93-1.06) | 0.98 (0.90-1.06) |
| Sex |  |  |  |  |  |
| Females | 51 (56.7) | 1.00 | 1.00 | 1.00 | 1.00 |
| Males | 39 (43.3) | 2.35 (0.91-6.07) | 2.32 (0.87-6.20) | 1.30 (0.63-2.69) | 1.41 (0.64-3.07) |
| Performance status |  |  |  |  |  |
| ≥90 | 51 (56.7) | 1.00 | 1.00 | 1.00 | 1.00 |
| <90 | 39 (43.3) | 2.18 (0.84-5.62) | 2.45 (0.79-7.60) | 2.02 (0.96-4.22) | 2.16 (0.88-5.30) |
| Extranodal disease |  |  |  |  |  |
| No | 46 (51.1) | 1.00 | 1.00 | 1.00 | 1.00 |
| Yes | 44 (48.9) | 1.37 (0.54-3.48) | 1.05 (0.34-3.24) | 1.54 (0.73-3.22) | 0.92 (0.36-2.36) |
| Ann Arbor stage |  |  |  |  |  |
| I-II | 55 (61.1) | 1.00 | 1.00 | 1.00 | 1.00 |
| III-IV | 35 (38.9) | 1.74 (0.69-4.39) | 1.11 (0.37-3.33) | 1.56 (0.75-3.25) | 1.18 (0.50-2.77) |
| Region of residence |  |  |  |  |  |
| Lima province | 41 (45.6) | 1.00 | 1.00 | 1.00 | 1.00 |
| Other provinces | 49 (54.4) | 1.11 (0.44-2.82) | 1.20 (0.43-3.29) | 2.09 (0.95-4.59) | 2.36 (0.98-5.70) |
| Frontline regimen |  |  |  |  |  |
| R-CHOP | 69 (77.5) | 1.00 | 1.00 | 1.00 | 1.00 |
| R-EPOCH/R-CHOEP | 10 (11.2) | 0.98 (0.22-4.30) | 1.71 (0.34-8.53) | 0.95 (0.28-3.16) | 1.43 (0.39-5.26) |
| CHOP/CHOEP | 8 (9.0) | 0.49 (0.07-3.74) | 0.51 (0.06-4.18) | 0.70 (0.17-2.99) | 1.21 (0.26-5.60) |
| FAB/LMB* | 2 (2.2) | 0.00 (-) | 0.00 (-) | 1.40 (0.19-10.39) | 1.20 (0.09-16.28) |

* Low counts yielded infinite values
