# Supplementary material for: Real-World Outcomes of Adolescents and Young Adults with Diffuse Large B-Cell Lymphoma: A Multicenter Retrospective Cohort Study
Source: J Adolesc Young Adult Oncol. 2024 Apr 2;13(2):323–30. doi: 10.1089/jayao.2023.0095 (PMC10998009; doi:10.1089/jayao.2023.0095)
Supplement: Supplemental data [file Suppl_FigS1.docx]

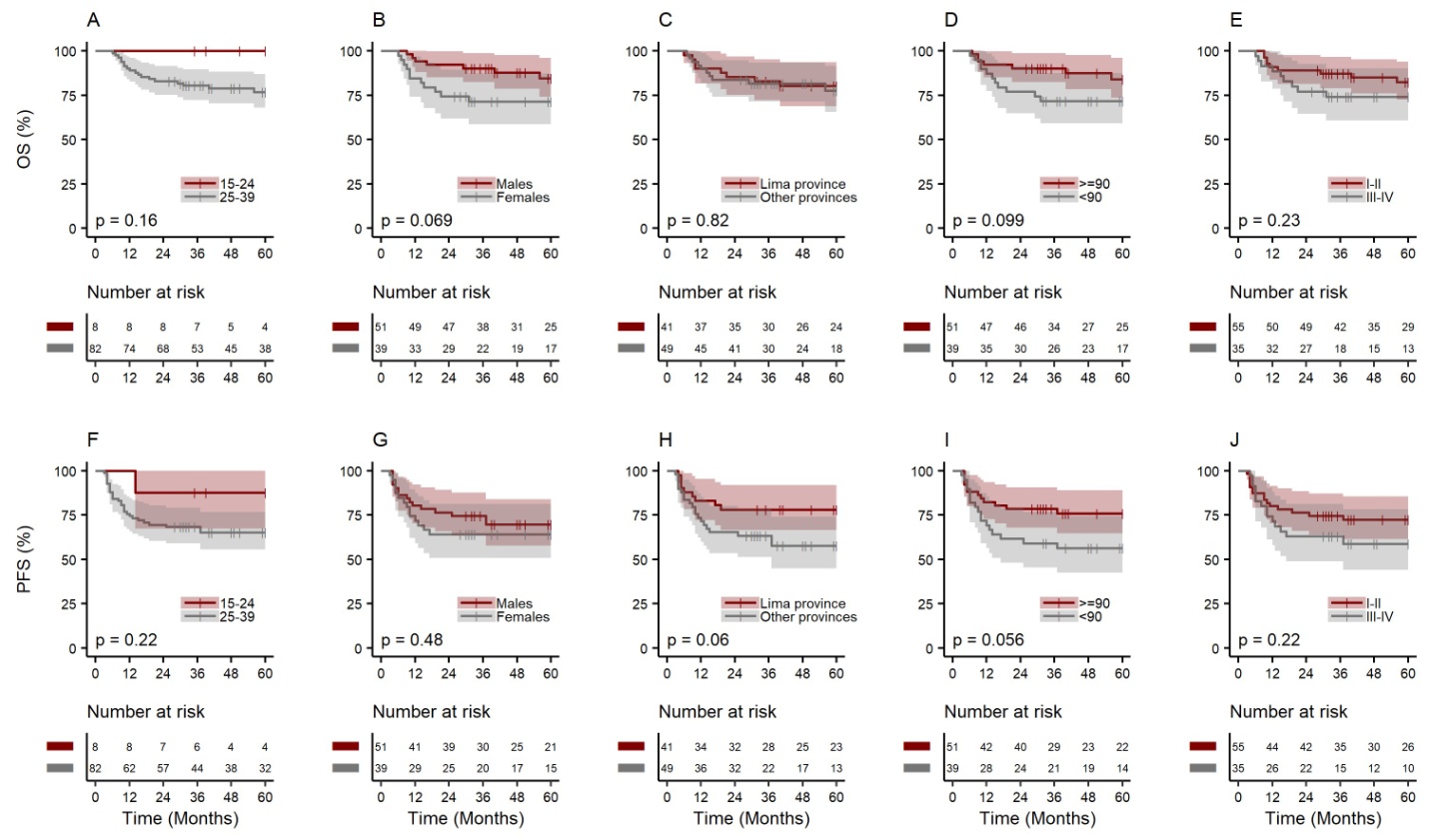


Supplementary Figure 1. Kaplan-Meier curves for OS and PFS rates by demographic and clinical factors among all AYAs with DLBCL.
