# Supplementary material for: Real-World Outcomes of Adolescents and Young Adults with Diffuse Large B-Cell Lymphoma: A Multicenter Retrospective Cohort Study
Source: J Adolesc Young Adult Oncol. 2024 Apr 2;13(2):323–30. doi: 10.1089/jayao.2023.0095 (PMC10998009; doi:10.1089/jayao.2023.0095)
Supplement: Supplemental data [file Suppl_TableS2.docx]

**Supplementary Table 2**. Clinical and treatment features of AYAs with R/R DLBCL.

| **Characteristics** | **No. (%)** |
| --- | --- |
| No. of patients | 29 |
| Median age at diagnosis, years | 33 |
| Age group |  |
| 15-24 | 1 (3) |
| 25-39 | 28 (97) |
| Males | 15 (52) |
| Performance status* |  |
| <90 | 17 (59) |
| ≥90 | 12 (41) |
| Nodal involvement | 24 (83) |
| Extranodal involvement | 16 (55) |
| B symptoms | 18 (62) |
| Clinical stage |  |
| I-II | 14 (48) |
| III-IV | 15 (52) |
| Frontline treatment response of the R/R group |  |
| CR | 9 (31) |
| PR | 13 (45) |
| PD | 7 (24) |
| Second line treatment | 24 (83) |
| Second-line regimen |  |
| R-ICE | 11(46) |
| R-ESHAP | 8 (34) |
| R-GEMOX | 1 (4) |
| R-GDP | 1 (4) |
| R-DHAP | 1 (4) |
| ICE | 2 (8) |
| GEMOX | 0 (-) |
| Unknown | 5 |
| HSCT after second line | 4 (17 ) |

* Performance status was evaluated by Lansky or Karnosfky scales.

Abbreviations: CR: complete response; PR: partial response; PD: progressive disease; R-ICE:  rituximab, ifosfamide, carboplatin and etoposide; R-ESHAP: rituximab, etoposide, methylprednisone, cytarabine, cisplatin; R-GEMOX; rituximab plus gemcitabine and oxaliplatin; R-GDP: rituximab, gemcitabine, dexamethasone. And cisplatin; R-DHAP: rituximab, dexamethasone, cytarabine, cisplatin; ICE: ifosfamide, carboplatin and etoposide; GEMOX; gemcitabine and oxaliplatin and HSCT: hematopoietic stem cell transplantation.
